# Thymic plasmacytoid dendritic cells are susceptible to productive HIV-1 infection and efficiently transfer R5 HIV-1 to thymocytes *in vitro*

**DOI:** 10.1186/1742-4690-8-43

**Published:** 2011-06-03

**Authors:** Vanessa A Evans, Luxshimi Lal, Ramesh Akkina, Ajantha Solomon, Edwina Wright, Sharon R Lewin, Paul U Cameron

**Affiliations:** 1 Monash University, Department of Medicine, Central and Eastern Clinical School, Alfred Campus, Commercial Rd., Melbourne, Victoria 3004, Australia; 2 Burnet Institute, Centre for Virology, Melbourne, Victoria 3004, Australia; 3 Colorado State University, Department of Microbiology, Immunology and Pathology, Fort Collins, CO 80523-1619, USA; 4 The Alfred Hospital, Infectious Diseases Unit, Commercial Rd., Melbourne, Victoria 3004, Australia; 5 Monash University, Department of Immunology, Central and Eastern Clinical School, Alfred Campus, Commercial Rd., Melbourne, Victoria 3004, Australia

## Abstract

**Background:**

HIV-1 infection of the thymus contributes to the defective regeneration and loss of CD4^+ ^T cells in HIV-1-infected individuals. As thymic dendritic cells (DC) are permissive to infection by HIV-1, we examined the ability of thymic DC to enhance infection of thymocytes which may contribute to the overall depletion of CD4^+ ^T cells. We compared productive infection in isolated human thymic and blood CD11c^+ ^myeloid DC (mDC) and CD123^+ ^plasmacytoid DC (pDC) using enhanced green fluorescent protein (EGFP) CCR5 (R5)-tropic NL(AD8) and CXCR4 (X4)-tropic NL4-3 HIV-1 reporter viruses. Transfer of productive HIV-1 infection from thymic mDC and pDC was determined by culturing these DC subsets either alone or with sorted thymocytes.

**Results:**

Productive infection was observed in both thymic pDC and mDC following exposure to R5 HIV-1 and X4 HIV-1. Thymic pDC were more frequently productively infected by both R5 and X4 HIV-1 than thymic mDC (p = 0.03; n = 6). Thymic pDC efficiently transferred productive R5 HIV-1 infection to both CD3^hi ^(p = 0.01; mean fold increase of 6.5; n = 6) and CD3^lo ^thymocytes (mean fold increase of 1.6; n = 2). In comparison, transfer of productive infection by thymic mDC was not observed for either X4 or R5 HIV-1.

**Conclusions:**

The capacity of thymic pDC to efficiently transfer R5 HIV-1 to both mature and immature thymocytes that are otherwise refractory to R5 virus may represent a pathway to early infection and impaired production of thymocytes and CD4^+ ^T cells in HIV-1-infected individuals.

## Background

The thymus is critical to CD4^+ ^T cell homeostasis and is the major source of naïve CD4^+ ^T cells throughout life [1-4]. HIV-1 can inhibit proliferation of immature thymocytes [5] and/or can directly infect CD4^+ ^thymocytes [6] leading to impaired production of CD4^+ ^T cells, which contributes to progressive CD4^+ ^T cell decline. Studies in both humanised mouse models [7-10] and human fetal thymic organ cultures [9] have shown thymocytes to be infected with both CCR5 (R5) and CXCR4 (X4)-tropic HIV-1. In comparison, single cell suspensions of thymocytes are relatively resistant to infection with R5 HIV-1 [11]. As CXCR4 is highly expressed on most thymocytes, while CCR5 is only expressed on a relatively small proportion of thymocytes [12], the mechanism by which thymocytes are infected with R5 virus remains unclear.

Dendritic cells (DC) in the thymus cluster closely with resident thymocytes and are permissive to HIV-1 infection [13,14]. Thymic DC can be broadly grouped into a major CD123^+ ^plasmacytoid (pDC) population and a smaller CD11c^+ ^myeloid (mDC) population [15-17]. While the function of thymic pDC remains unknown, it has been suggested that they play a role in protecting the thymus against viral infection [15,18] and/or influence positive selection of thymocytes [19,20] through the secretion of IFN-alpha. Following HIV-1 infection *in vitro*, IFN-alpha is produced by thymic pDC but does not inhibit viral replication within thymocytes [18]. Thymic pDC may instead play the role of a 'Trojan horse' [14]. We hypothesised that HIV-1-infected thymic DC facilitate infection of thymocytes with R5 virus following cell-to-cell contact in a similar fashion to how blood pDC and mDC facilitate infection of CD4^+ ^T cells isolated from blood [21]. Here we show that thymic pDC are permissive to high levels of both productive R5 and X4 HIV-1 infection. Furthermore, we demonstrate transfer of productive R5 HIV-1 infection from thymic pDC to CD3^hi ^and CD3^lo ^thymocytes. These results demonstrate the importance of thymic pDC in facilitating infection of immature and mature thymocytes.

## Results

### Three subpopulations of DC exist in the human thymus

**Figure 1:**
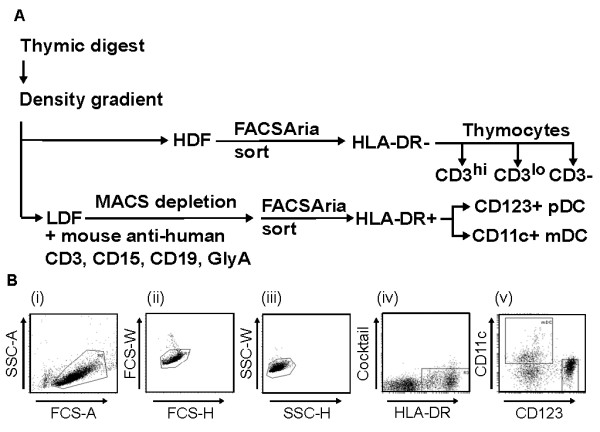
**Strategy for the isolation and sorting of thymic pDC and mDC**. (A) Thymus tissue was digested to create a single cell suspension. Using Nycodenz density centrifugation cells were then divided into a low-density (LDF) and a high-density fraction (HDF). Thymocyte subpopulations were sorted from the HDF. DC were enriched from the LDF by magnetic bead depletion (MACS) and high speed flow cytometric cell sorting (FACSAria). (B) Gating strategy for sorting thymic DC; (i) Viable cells were selected by live gating using forward (FSC) and side scatter (SSC); (ii and iii) Doublets were excluded based on FSC-width (W), -height (H) and SSC-W, H; (iv) HLA-DR-positive, cocktail (CD3, CD15, CD19, GlyA)-negative cells were selected and (v) CD123^+ ^(pDC) and CD11c^+ ^(mDC) populations were sorted. On average, the recovery of isolated DC was 3 × 10^5 ^pDC and 2 × 10^5 ^mDC per 10^9 ^total thymic cells. The purity for pDC and mDC was always greater than 98%.

**Figure 2:**
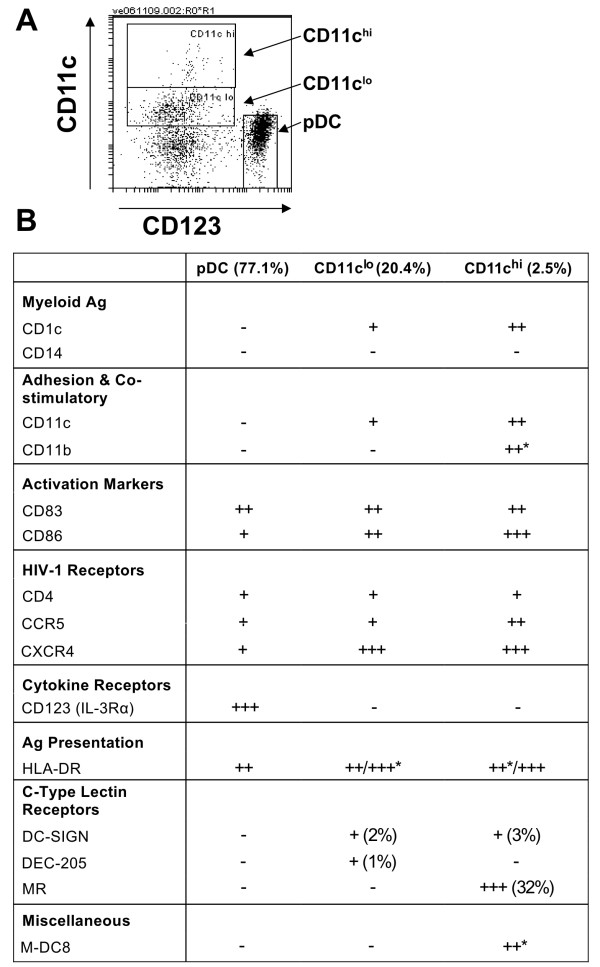
**Phenotypic identification of thymic DC**. (A) DC were gated based on CD123, CD11c^lo ^or CD11c^hi ^expression. (B) A major plasmacytoid (pDC; HLA-DR^int^CD123^hi^CD11c^-^; 77.1%); a smaller myeloid (HLA-DR^int/hi^CD123^-^CD11c^lo^; 20.4%); and a minor myeloid-related M-DC8^+ ^(HLA-DR^int/hi^CD123^-^CD11c^hi^M-DC8^+^; 2.5%) DC population were identified. Expression of myeloid antigens (CD1c and CD14), adhesion and co-stimulatory molecules (CD11c and CD11b), HIV-1 receptors (CD4, CXCR4 and CCR5), maturation markers (CD83 and CD86), C-type lectin receptors (DC-SIGN, DEC-205 and MR), CD123, HLA-DR and M-DC8 was determined. Mean fluorescence intensity (MFI) > 2000 (+++), MFI 500-2000 (++), MFI 60-500 (+) or absent (-). * ≥ 50% of population positive (representative data from 2-3 separate experiments).

We first characterised the frequency and phenotype of DC in human thymus and then quantified expression on these DC subsets of the HIV-1 co-receptors CXCR4 and CCR5, as well as C-type lectins known to be important for HIV-1 transfer. We used methods of isolation similar to previous studies of thymic DC [15-17] and identified three predominant thymic DC subpopulations in the human thymus, based on the expression of the known DC surface markers CD11c, CD123 and HLA-DR (Figure 1A and 1B). These populations were HLA-DR^int^CD123^+^CD11c^-^, HLA-DR^int/hi^CD123^-^CD11c^lo^, and HLA-DR^int/hi^CD123^-^CD11c^hi ^(Figure 2A and 2B). CD14 expression was determined to eliminate the possibility of monocyte contamination; all DC subsets were negative for CD14. The HLA-DR^int^CD123^+^CD11c^- ^pDC represented the predominant population of DC in the thymus (77% of total DC). This was in contrast to human blood, where mDC were the major subset of DC (68% of total DC; data not shown). Like blood pDC, thymic pDC expressed high levels of the cytokine receptor CD123, while they lacked both expression of the myeloid marker CD1c and the adhesion and co-stimulatory molecules CD11b and CD11c. A smaller population of myeloid-related CD123^-^CD11c^lo ^DC was also identified (20% of total DC). These cells were heterogeneous for HLA-DR expression, with 72% of cells expressing high levels of HLA-DR, while the remaining cells expressed intermediate levels (Figure 2B).

A third minor subpopulation of CD123^-^CD11c^hi ^DC was also observed (3% of total DC), with the majority of cells (66% of CD11c^hi^) expressing high levels of HLA-DR and CD86. Additionally, 67% of CD11c^hi ^DC expressed CD11b and 88% of the CD11c^hi ^DC expressed the P-selectin glycoprotein ligand 1 M-DC8, a marker shared with the CD16^+ ^DC subset found in blood [22]. The M-DC8^+ ^thymic DC did not express CD16 (not shown). These data demonstrated that the major DC population in the thymus is pDC, followed by mDC and then a minor unique population of CD11c^hi ^DC that also express M-DC8.

We next determined the expression of surface receptors required for HIV-1 entry on each thymic DC subset. CD4, CCR5 and CXCR4 were detected in all three thymic DC subsets. In contrast, the C-type lectin receptors DC-SIGN, DEC-205 and MR, which have been shown to play a role in the transfer of HIV-1, were only detected on a small proportion of mDC/CD11c^hi ^DC (Figure 2B).

### Thymic DC show a differential susceptibility to productive HIV-1 infection

**Figure 3:**
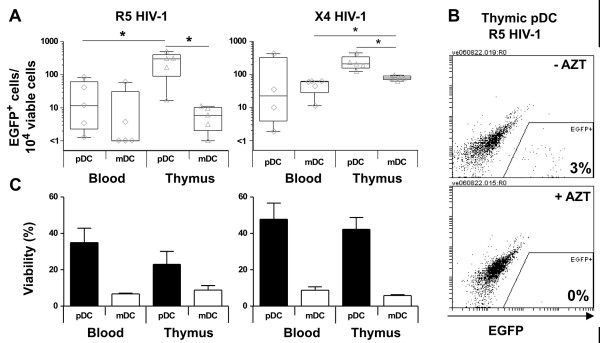
**Frequency of R5 and X4 HIV-1 infection in blood and thymic DC**. (A) Plasmacytoid DC (pDC) and myeloid DC (mDC) were isolated from blood and thymus tissue. Cells were exposed to either R5 (NL(AD8)-nef/EGFP) or X4 HIV-1 (NL4-3-nef/EGFP). The number of EGFP^+ ^cells was determined by flow cytometry 5 days post-infection. Each symbol represents the mean of duplicate experiments from a single donor. The edges of the boxes represent the 25 and 75 percentiles, the horizontal line in the box is the median and the whiskers extend to the minimum and maximum data points. * indicates a p value of < 0.05 as determined by the Wilcoxon signed rank test. (B) In some experiments the DC were pre-treated with AZT (0.1 μM) prior to infection. Representative results form 4 experiments are shown. (C) DC viability was assessed by flow cytometry following 5 days of culture and expressed as a proportion of live cells over total cells. Each column represents the mean of 5-6 experiments ± SEM.

We then infected freshly isolated CD123^+ ^pDC and CD11c^+ ^mDC (comprising both the M-DC8^+ ^and M-DC8^- ^DC subsets) from unmatched human blood and thymus (n = 12). The DC populations were mock infected with media alone or infected with either X4 or R5 viruses containing a deletion in the *nef *gene (-nef) and replaced with enhanced green fluorescent protein (EGFP) [pDRNL4-3-nef/EGFP or pDRNL(AD8)-nef/EGFP respectively] (Figure 3A). Both thymic DC subsets were productively infected by HIV-1; however, the frequency of both R5 and X4 HIV-1-infected cells was significantly greater in thymic pDC compared to donor-matched thymic mDC (p = 0.03; n = 6; Figure 3A). Following infection with R5 HIV-1, the median number of EGFP^+ ^cells was higher in thymic pDC (233 events/10^4 ^viable cells; n = 6) than in thymic mDC (6 events/10^4 ^viable cells; p = 0.03). Thymic pDC were infected with X4 HIV-1 (207 events/10^4 ^viable cells) at a similar frequency to R5 HIV-1. In contrast, thymic mDC were more frequently infected with X4 (78 events/10^4 ^viable cells) than R5 HIV-1 (6 events/10^4 ^viable cells; p = 0.004). Furthermore, no EGFP^+ ^cells were present following infection of either pDC or mDC in the presence of azidothymidine (AZT), indicating that EGFP expression represented the production of new virus proteins within the DC during a spreading infection (Figure 3B).

In comparison to thymic DC cultures, we did not detect a statistically significant difference in EGFP^+ ^cells in blood pDC compared to blood mDC following infection with both R5 (10 and 1 events/10^4 ^viable cells respectively; p = 0.22) and X4 HIV-1 (22 and 59 events/10^4 ^viable cells respectively; p = 0.44). Blood pDC were equally infected by X4 and R5 HIV-1 (p = 0.38). In contrast, blood mDC, like thymic mDC, were more permissive to X4 than R5 virus (p = 0.01). Finally, when we compared thymic and blood DC, we found that thymic mDC were significantly more permissive to productive infection by X4 HIV-1 compared with mDC purified from blood (p = 0.004). While thymic pDC were significantly more permissive to R5 HIV-1 than blood pDC (p = 0.01).

Although DC were cultured with both IL-3 and GM-CSF, at concentrations that maintain cell viability and allow for some maturation of DC [23,24], thymic and blood mDC had a lower viability than thymic and blood pDC (as determined by flow cytometry live gating analysis using forward and side scatter parameters) following 5 days of cell culture and infection with either R5 or X4 virus, (p = 0.06; Figure 3C). However, when the number of total EGFP^+ ^cells was analysed rather than the number of viable EGFP^+ ^cells, our observations remained unchanged, suggesting that the low viability of mDC did not contribute to their differential susceptibility to infection when compared to pDC.

### Thymic pDC transfer R5 but not X4 HIV-1 to mature single-positive thymocytes

**Figure 4:**
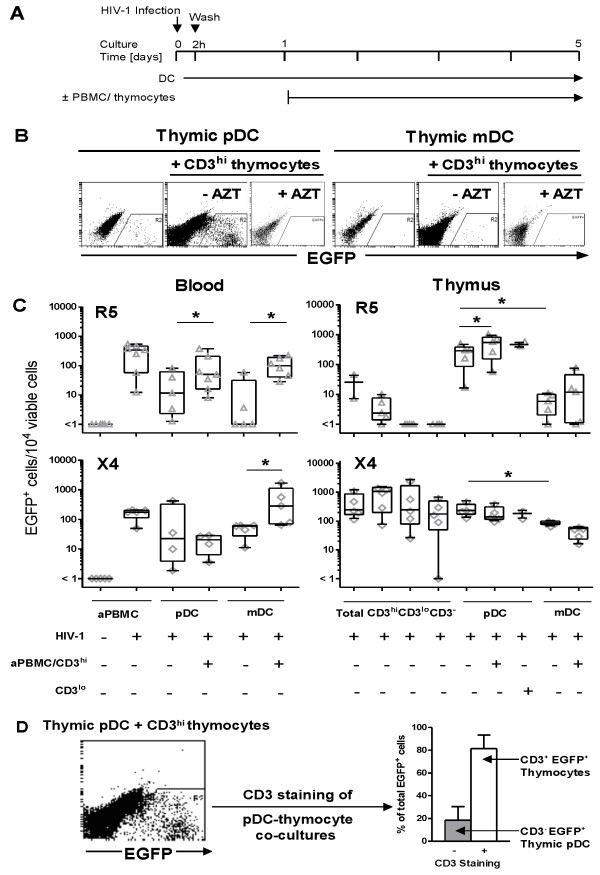
**DC Transfer of R5 and X4 HIV-1: Blood versus thymus**. (A) Method used to detect transfer of HIV-1. DC were infected with R5 (NL(AD8)-nef/EGFP) or X4 (NL4-3-nef/EGFP) HIV-1. Mock PBMC and thymocytes were cultured in parallel. DC were either cultured alone for the duration of the culture period (5 days), or at 24 h post-infection mock PBMC were added to the blood DC and mock CD3^hi^/CD3^lo ^thymocytes were added to the thymic DC at a 1:1 ratio. (B) Results from a single experiment demonstrate the gating strategy to determine the number of EGFP^+ ^cells following R5 infection of thymic pDC (left panels) and mDC (right panels) cultured alone or in the presence of CD3^hi ^thymocytes. In some experiments, thymocytes were treated with 0.1 μM AZT prior to co-culture with DC. Representative plots from 4 donors are shown. (C) Blood (left hand panels) and thymic (right hand panels) DC infected with either R5 (upper row) or X4 (lower row) HIV-1. Each symbol represents the mean of duplicate experiments from a single donor. The edges of the boxes are the 25 and 75 percentiles, the horizontal line in the box is the median and the whiskers extend to the minimum and maximum data points. * indicates a p value of < 0.05 as determined by the Wilcoxon signed rank test. The results for 5-6 donors are shown. (D) Thymic pDC were infected with R5 HIV-1. At 24 h post-infection, uninfected CD3hi thymocytes were added to the pDC at a 1:1 ratio. Culture was continued for 4 days, at which time the pDC-thymocyte co-cultures were labelled with CD3, and the number of EGFP^+^CD3^+ ^thymocytes was determined by flow cytometry. Columns represent the mean of 2 experiments ± SEM.

We next determined whether HIV-1 infection of thymic DC facilitated infection of thymocytes. Human blood and thymic DC were infected with R5 or X4 HIV-1 for 2 h and then washed to remove any unbound virus. The HIV-1-exposed blood and thymic DC were then cultured for 24 h before adding an equal number of uninfected autologous peripheral blood mononuclear cells (PBMC) or CD3^hi ^thymocytes respectively. Culture was continued for 4 days before analysis by flow cytometry (Figure 4A). We assumed that any increase in the proportion of EGFP^+ ^cells following infection of the co-cultures, compared to infection of DC cultured alone, would indicate viral transfer from the DC to either the PBMC or CD3^hi ^thymocytes (Figure 4B). If transfer of virus did not occur, then the proportion of EGFP^+ ^cells would be expected to decrease due to the dilution of DC with PBMC or thymocytes.

As previously reported [11], thymocytes in single cell suspension were productively infected by X4 but not R5 HIV-1 infection (Figure 4C). Following exposure to X4 HIV-1, the highest median level of EGFP^+ ^cells was detected in the mature CD3^hi ^thymocytes (923 events/10^4 ^viable cells), with fewer EGFP^+ ^cells observed in the less mature CD3^lo ^and CD3^- ^thymocytes (217 and 156 events/10^4 ^viable cells respectively; p = 0.03). In comparison, following exposure to R5 HIV-1 we only detected a very low number of EGFP^+ ^cells in the mature CD3^hi ^thymocytes (2 events/10^4 ^viable cells) and no infection in the CD3^lo ^or CD3^- ^thymocytes. This data confirmed previous findings of relative resistance of thymocytes to R5 infection *in vitro*.

In this culture system, only R5 exposed thymic pDC were able to transfer productive infection to CD3^hi ^thymocytes. The number of EGFP^+ ^cells observed in the thymic pDC-thymocyte co-cultures was significantly higher when compared to the pDC cultured alone (mean fold increase of 6.5; p = 0.01; Figure 4B and 4C). Transfer was confirmed in the pDC-CD3^hi ^thymocyte co-cultures by demonstrating that the majority of EGFP^+ ^cells were also positive for CD3 expression (mean of 70%; n = 2; Figure 4D). In two experiments, CD3^lo ^thymocytes were added to the pDC 24 h post infection, and transfer of R5 virus to the CD3^lo ^thymocytes was also observed, although transfer was less efficient (mean fold increase of 1.65) than that observed to the CD3^hi ^thymocytes. Thymic pDC did not, however, transfer productive X4 HIV-1 infection to CD3^hi ^thymocytes as the proportion of EGFP+ cells observed in the thymic pDC-thymocyte co-cultures was lower when compared to the pDC cultured alone (125 and 207 events/104 viable cells respectively; Figure 4C).

In comparison, thymic mDC did not transfer productive R5 or X4 HIV-1 to CD3^hi ^thymocytes. The number of EGFP^+ ^cells was similar following R5 and X4 HIV-1 infection in the mDC cultured alone (6 and 78 events/10^4 ^viable cells respectively) when compared to those cultured with CD3^hi ^thymocytes (8 and 50 events/10^4 ^viable cells respectively; Figure 4C).

In order to determine differences in the capacity of DC from thymus and other sites to transfer HIV-1, we next examined whether human blood mDC and pDC transferred HIV-1 to unstimulated PBMC. Blood mDC were shown to efficiently transfer both R5 (mean fold increase of 65.9; p = 0.02; Figure 4C) and X4 HIV-1 (mean fold increase of 15.1; p = 0.03; Figure 4C) to PBMC while blood pDC only transferred R5 HIV-1 (mean fold increase of 4; p = 0.03; Figure 4C). Additionally, blood mDC were significantly more efficient at transferring R5 HIV-1 to PBMC compared to blood pDC (p = 0.03).

Taken together, these experiments demonstrated that human thymic and blood DC differed in their susceptibility to X4 and R5 HIV-1 and also had a different capacity for transfer of HIV-1. Importantly, thymic pDC were able to transfer R5 virus to both CD3^hi ^and CD3^lo ^thymocytes, and may explain how thymocytes are infected with R5 virus.

### pDC are located within the cortex and medulla in uninfected and HIV-1-infected thymus tissue

**Figure 5:**
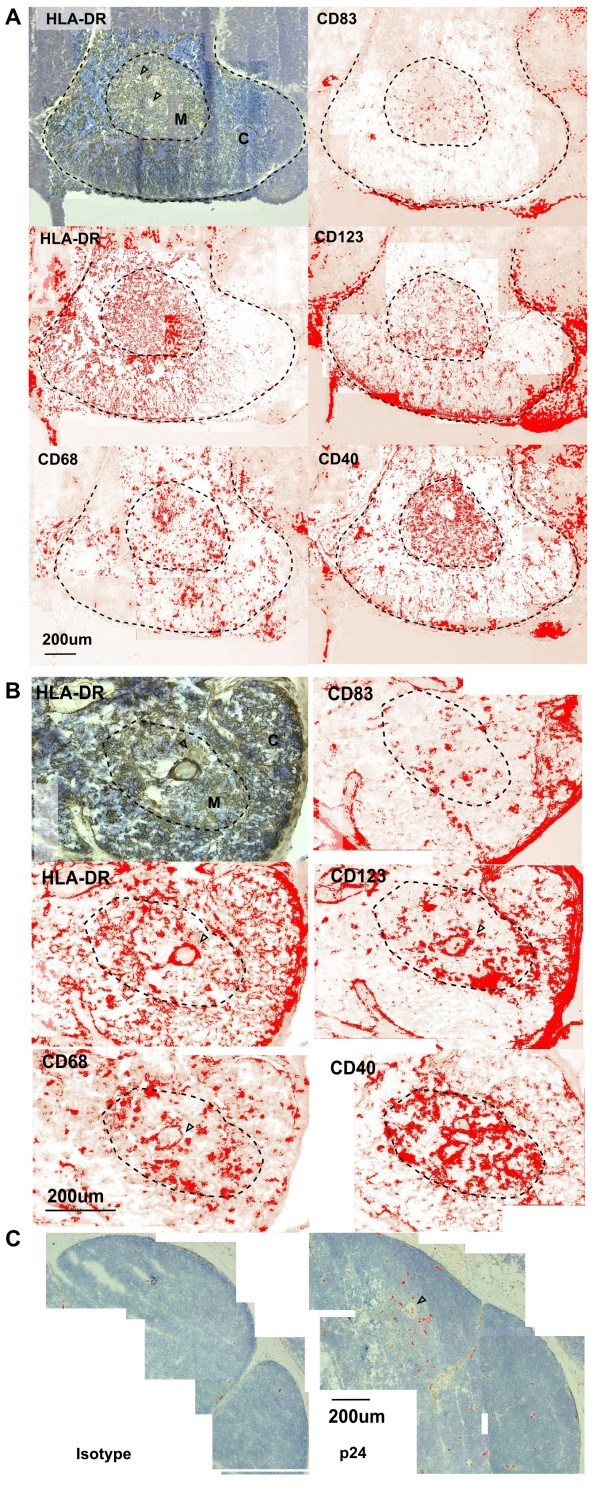
**Localisation of CD123^+ ^pDC in uninfected and HIV-1-infected thymus tissue**. Immunohistochemistry was performed on fresh frozen thymus sections. Tissue sections were examined using an Olympus BX50 microscope (Olympus, Centre Valley, PA) and images captured using a ProSeries 3CCD camera (SciTech). Sections were imaged with a 20x objective and montages were created and composite images were colour deconvoluted using H-DAB filter in ImageJ (National Institutes of Health, Bethesda, MD). The threshold was set for 3,3'-Diaminobenzidine-positive cells (red). The medulla (M) was identified by the presence of Hassall's corpuscles (HC, open arrow head), increased HLA-DR expression, high CD40 expression and the presence of CD83^+ ^medullary DC. (A) In thymus tissue from uninfected human donors the medulla and cortex [C] included cells expressing high levels of CD123. (B) The distribution of CD123^+ ^cells in thymic grafts from day 7 HIV-1_BaL_-infected SCID-hu-thy-liv mice was similar to normal thymus tissue. (C) Sections were labelled with isotype control or p24 antibody and processed for immunohistochemistry using DAB. The cortical regions were imaged at an original magnification of 20x and a montage created before processing for colour deconvolution using ImageJ. The threshold was set on the DAB channel, particles analysed using ImageJ, and particle masks were then overlayed onto the original montaged image. Scale bar is 200 μm.

Given that thymocytes of different maturation are found in the cortex and medulla, and our findings demonstrated that thymic pDC were able to transfer productive R5 HIV-1 infection to thymocytes, we next examined the distribution of pDC within the human thymus using antibodies to CD123, HLA-DR, CD68, CD83 and CD40 (Figure 5A). The medulla was identified by the presence of Hassall's corpuscles, increased HLA-DR expression, high CD40 expression and the presence of CD83^+ ^medullary DC. We then identified cells that were CD123^+ ^and HLA-DR^+ ^(Figure 5A), indicative of pDC, in both the cortex and the medulla of uninfected human thymus sections. To determine the effect of HIV-1 infection on pDC distribution in the thymus, and because we were unable to access thymus from HIV-1-infected patients, we examined the distribution of CD123^+ ^cells in thymus tissue collected from severe combined immunodeficiency (SCID) mice transplanted with human fetal liver and thymic tissues (SCID-hu-thy-liv) infected with R5 HIV-1_BaL_. At day 7 post infection, p24+ cells were visible in both the cortex and medulla (Figure 5C), and CD123^+ ^cells were found to have a similar distribution in thymus tissue from these infected animals (Figure 5B) when compared to uninfected thymus tissue (Figure 5A). These studies, therefore, confirmed that pDC were present in both the cortex and medulla in human thymus in the presence and absence of HIV-1 infection *in vivo*.

## Discussion

The thymus plays a critical role in CD4^+ ^T cell homeostasis. Thus, it is important to understand how thymocytes and other thymic cells are infected with HIV-1. Thymocytes express low levels of CCR5 and are permissive to infection with R5 HIV-1 *in vivo *[8,25], but are relatively resistant to R5 HIV-1 infection as single cell suspensions. In this study we provide a mechanism for how R5 HIV-1 infects thymocytes by demonstrating that thymic pDC were able to transfer productive R5 HIV-1 infection to both CD3^hi ^and CD3^lo ^thymocytes. The efficient transfer of R5 HIV-1 by thymic pDC to thymocytes and their proximity to immature thymocytes within the thymic cortex may provide a pathway for R5 HIV-1 infection of both mature and immature thymocytes.

Previous studies have demonstrated the ability of blood and tissue DC to transfer HIV-1 infection to CD4^+ ^T cells [21,26-28]. In monocyte-derived DC (MDDC), this transfer occurs in two phases. In the first phase, transfer largely occurs via trans-infection, which is followed by rapid decay of the virus. The second phase includes transfer of virus from productively infected DC to CD4^+ ^T cells [29]. Tissue DC isolated from tonsils and skin (Langerhans cells) can transfer both R5 and X4 HIV-1 to CD4^+ ^T cells [13,30]; however, there have not been any studies to determine whether thymic DC possess a similar ability. We found that thymic pDC efficiently transferred productive R5 but not X4 HIV-1 to mature CD3^hi ^thymocytes. While thymic pDC only transferred R5 HIV-1, we observed high levels of both R5 and X4 HIV-1 infection of thymic pDC. Therefore, it is unlikely that the level of HIV-1 infection played a key role in the ability of thymic pDC to transfer R5 HIV-1 infection. Thymic pDC had a similar expression of CCR5 and CXCR4 and lacked the C-type lectin receptors more commonly associated with DC transfer of HIV-1. A potential explanation for the differences in transfer of R5 and X4 HIV-1, by thymic pDC, may be related to DC-thymocyte signalling. During clustering with thymic pDC, the creation of an immunological synapse leads to partial activation of the thymocytes that then allows for transfer of R5 but not X4 HIV-1. A similar mechanism has previously been described in MDDC [31,32].

Unlike HIV-1 infection of blood DC, we observed a significantly higher number of EGFP^+ ^cells in thymic pDC compared to thymic mDC following exposure to both R5 and X4 HIV-1 (Figure 3A). This is similar to the findings by Schmitt *et al. *who detected high levels of p24 following both R5 and X4 HIV-1 infection of thymic pDC, but failed to detect infection in the thymic CD11c^+^CD14^- ^mDC population [14]. These observations were independent of the levels of CCR5 and CXCR4, which were comparable across the two DC subsets [14]. Another study has shown that the fusion efficiency of R5 viruses declines as DC mature and CCR5 expression decreases, while X4 fusion efficiency does not change with DC maturation [33]. While we did not observe reduced CCR5 expression in thymic mDC, we demonstrated that the majority (71%) of thymic CD11c^+ ^mDC expressed high levels of HLA-DR and CD86 (Figure 2), indicating a mature phenotype. This was in contrast to the thymic pDC subset that expressed intermediate levels of HLA-DR and lacked CD86. It is possible that the less mature state of the thymic pDC may explain the higher levels of productive R5 HIV-1 infection in these cells compared to thymic mDC.

Thymic DC are a heterogenous population of cells, with up to 5 populations previously described [15-17,34]. Consistent with the findings of another group [15], we identified a major HLA-DR^int^CD11c^- ^pDC population, a smaller HLA-DR^int^CD11c^+ ^mDC population and a minor CD11c^hi ^DC population within the human thymus (Figure 2B). High expression of the P-selectin glycoprotein ligand 1 (PSGL-1) M-DC8 on a subpopulation (88%) of the CD11c^hi ^thymic DC is a novel observation, however, this population is unlikely to significantly transfer HIV-1 to thymocytes as these cells were included together with the thymic mDC, that did not transfer virus. Instead, thymic M-DC8^+ ^DC may contribute to the establishment of central tolerance, as PSGL-1 has previously been shown to play a role in the homing of antigen-bearing DC to the thymus [35].

Using immunohistochemistry we identified both CD123^+ ^and HLA-DR^+ ^cells in the cortex and medulla of uninfected thymus tissue (Figure 5A). These results were consistent with those of previous studies that have shown pDC in the cortex, in addition to the medulla and cortico-medullary junction where the majority of other DC subsets, including all the mature and mDC, are localised [16,17,36]. Infection of the thymus with R5 HIV-1_BaL _did not affect the distribution of the CD123^+ ^or HLA-DR^+ ^cells (Figure 5B). X4 HIV-1 infection of immature (CD3^-^CD4^+/lo^CD8^-^) thymocytes located in the thymic cortex has been shown to prevent their maturation into mature functional CD4^+ ^T cells *in vitro *[37,38]. Given the high susceptibility of thymic pDC to productive R5 HIV-1 infection, and their ability to transfer this infection to both immature and mature thymocytes, it is possible that the transfer of HIV-1 to immature thymocytes located within the cortex could prevent thymocyte maturation. Subsequently, a significant decrease in all thymocyte subpopulations may result, thus contributing to the overall depletion of CD4^+ ^T cells in HIV-1 infection [9].

Some limitations of the present study should be recognised. EGFP reporter viruses are important tools for evaluating productive HIV-1 infection in rare cells, such as DC, because EGFP enables the identification of a single infected cell. To construct the EGFP reporter viruses, EGFP was inserted into the HIV-1 *nef *gene and consequently, the *nef *gene was non-functional. Nef has previously been shown to boost HIV-1 replication in tonsil tissue [39]. Therefore, it is possible similar experiments that utilise *nef *competent strains may result in higher levels of productive infection of both thymic and blood DC. Additionally, future studies would benefit from the use of other R5 and X4 strains, including primary HIV-1 isolates, to confirm that our findings are relevant to a range of both laboratory and clinical isolates.

Limited production of CD4^+ ^T cells and delayed recovery of thymus function following treatment of HIV-1 infection are significant problems even with the availability of highly active antiretroviral therapy. In this study we demonstrated that thymic DC are a unique population, differing from blood subsets, and that thymic pDC are highly permissive to HIV-1 infection and efficiently transfer R5 HIV-1 to mature and immature thymocytes. Understanding transfer of HIV-1 from thymic DC to thymocytes may provide novel approaches to improve thymic output in HIV-1 infected patients.

## Conclusions

We have shown that the predominant thymic pDC subpopulation differs from thymic mDC in their greater ability to support replication of both X4 (NL4-3) and R5 (NL(AD8))-tropic strains of HIV-1. In addition, NL(AD8) replicated at higher levels in thymic pDC compared with blood pDC. Thymic pDC but not mDC were able to efficiently transfer NL(AD8) infection to both CD3^hi ^and CD3^lo ^thymocytes. Thus pDC provide a possible pathway for R5 HIV-1 infection of thymocytes and may contribute to the changes in thymic output seen in HIV-1 infection.

## Methods

### Thymus

Normal human thymus samples were discarded tissue from children (age range, 2 days to 7 years, n = 15) undergoing corrective cardiovascular surgery (Royal Children's Hospital, Melbourne, Australia) and were obtained with informed consent and under institutional guidelines. All experiments, excluding the immunohistochemistry, were conducted with this tissue. Infected thymus tissue for immunohistochemistry was obtained from HIV-1_BaL_-infected SCID-hu-thy-liv mice transplanted with human fetal liver and thymic tissue [40] (kindly supplied by Ramesh Akkina, Colorado State University, Fort Collins, USA). Infection of the SCID-hu-thy-liv thymus tissue was quantified in tissue digests by real time PCR (iCycler; Biorad, Hercules, CA) using previously described methods to detect full length HIV-1 DNA with primers specific for LTR and Gag [41].

### Thymocyte purification

Connective tissue was dissected from the human thymus samples and the thymus tissue disrupted with a scalpel blade prior to incubation with collagenase (1 mg mL^-1^, type II; Worthington Biochemical Corporation, Lakewood, NJ) and DNase (0.02 mg mL^-1^, grade II bovine pancreatic DNaseI; Worthington, Lakewood, NJ) in RPMI-1640 media (Gilbco/Invitrogen, Grand Island, NY) supplemented with 2% heat inactivated cosmic calf serum (HyClone, Logan, UT). Incubation was continued for 30 min at 37°C with intermittent agitation followed by 5 min at room temperature with constant agitation. To disrupt T cell-DC complexes, 100 mM EDTA was added (10 mM final concentration) to the digest, and incubation with agitation was continued for 5 min. The suspension was then passed through a nylon mesh to remove any remaining aggregates and/or stromal material. The resulting single cell suspension was subjected to Nycodenz (Axis-shield, Dundee, Scotland) density gradient centrifugation as previously described [17], with the exception that cells were resuspended in Nycodenz at a density of 1.070 g/mL, rather than 1.068 g/mL, as we found that this gave a greater DC yield. A low-density fraction (LDF) containing DC and a high-density fraction (HDF) were recovered. Immature double-negative (CD3^-^CD4^-^CD8^-^), double-positive (CD3^lo^CD4^+^CD8^+^) and mature single-positive (CD3^+^CD4^+^CD8^- ^or CD3^+^CD4^-^CD8^+^) thymocytes were isolated from the HDF using the monoclonal antibodies (mAbs); anti-HLA-DR-allophycocyanin-cychrome-7 (APC-Cy-7), anti-CD3-phycoerythrin (PE; BD Biosciences, Bedford, MA) and FACSAria cell sorting (BD Biosciences, Bedford, MA).

### Phenotypic analysis of thymic DC subsets

Phenotypic analysis was performed on the enriched thymic DC population recovered from the Nycodenz LDF. Cells were immunostained with labelled mouse mAbs and incubated for 25 min at 4°C. The mAbs included anti-CD11c-APC, anti-CD123-PE, anti-HLA-DR-PE/ Peridinin Chlorophyll Protein Complex (perCP)/ APC-Cy7, anti-CD14-PE, anti-CD3-fluorescein isothiocyanate (FITC), anti-CD4 perCP, anti-CCR5 FITC, anti-CXCR4 PE and APC (BD Biosciences, San Jose, CA), anti-CD1c-FITC (Biosource International, Camarillo, CA), anti-CD83 PE, anti-CD86 APC, anti-DC-SIGN PE, anti-DEC-205 perCP-Cy5, anti-MR APC (Biolegend, San Diego, CA), and anti-M-DC8 (kindly supplied by Knut Schakel; Institute of Immunology, Technical University of Dresden, Germany). Cells labelled with anti-M-DC8 were washed and incubated with goat anti-mouse IgM-biotin (Chemicon, Boronia, Australia) for 20 min at 4°C and finally washed and incubated with streptavidin-APC (Becton Dickinson, Franklin Lakes, NJ, USA) for 25 min at 4°C.

### Blood and thymic DC purification

For thymic DC purification, LDF cells were immunodepleted by magnetic cell sorting (Miltenyi Biotec, Bergisch Gladbach, Germany) using a cocktail of mAbs; anti-CD3 (OKT3), anti-CD15 (WEMG.I), anti-glycophorin A (GlyA; 10FM.N) and anti-CD19 (FMC63; a kind gift from Heddy Zola, Flinders Medical Centre, Adelaide, Australia), and anti-mouse IgG-coated magnetic microbeads (Miltenyi Biotec). For blood DC purification, PBMC isolated over Ficoll Hypaque gradients (Pharmacia, Uppsala, Sweden) from fresh buffy coats (Australian Red Cross Blood Service, Melbourne, Australia) were immunodepleted using the mAbs; anti-CD3 (OKT3), anti-CD11b (OKM1), anti-CD19 (FMC63) and anti-GlyA (10FM.N). The DC enriched populations were immunostained with sheep anti-mouse-FITC (Chemicon, Boronia, Australia) to identify any remaining cocktail-positive cells. After blocking with 10% normal mouse serum (Sigma, St. Louis, MO), cells were incubated with the mAbs; anti-CD11c-APC, anti-CD123-PE and anti-HLA-DR-APC-Cy7 (BD Biosciences). Using a FACSAria (BD Biosciences) we were able to sort two DC subpopulations by gating on total HLA-DR^+ ^cells, in order to exclude any contaminating basophils/mast cells/natural killer cells, and then either CD11c^+ ^mDC or CD123^+ ^pDC. The number of isolated DC did not correlate with the thymus donor age and on average the recovery was 3 × 10^5 ^pDCs and 2 × 10^5 ^mDCs per 10^9 ^total thymic cells. The purity of sorted cells was always greater than 98% upon reanalysis (Figure 1).

### Preparation and characterisation of HIV-1 stocks

HIV-1 viruses were generated by transfection of 293T cells with either X4 or R5 viruses [pDRNL4-3-nef/EGFP or pDRNL(AD8)-nef/EGFP respectively] (kindly supplied by Damien Purcell, The University of Melbourne, Melbourne, Australia). Supernatants were centrifuged, filtered through 0.45 μm pore-size filters, concentrated by ultra-centrifugation over a 20% sucrose gradient and stored at -80°C. The 50% tissue culture infective doses of the virus stocks was evaluated by limiting dilution on PHA (10 μg/mL; Murex, Kent, UK) stimulated PBMCs.

### Infection with and transfer of HIV-1

PHA-stimulated PBMC (aPBMC; positive control for productive infection), unstimulated PBMC, thymocytes and DC subpopulations were either mock infected with media alone or infected with viral supernatants at a multiplicity of infection of 0.1 at 37°C in RC-10 (RPMI-1640 supplemented with 10% (vol/vol) cosmic calf serum, 100 U/mL penicillin, 100 μg/mL streptomycin, 2.9 mg/mL L-glutamine (Gilbco/Invitrogen, Grand Island, NY)). Following 2 h of culture, the cells were washed thoroughly to remove unbound virus. Cells were cultured at 37°C in round-bottom 96-well microtitre plates at a concentration of 10^5 ^cells/100 μL RC-10. Thymocytes and PBMC were cultured with IL-2 (10U/mL; Roche Diagnostics, Indianapolis, IN), while DC were cultured with IL-3 (10 ng/mL; R&D Systems Inc, Minneapolis, MN) and GM-CSF (40 ng/mL; R&D Systems Inc), which have previously been reported to increase DC survival [23]. In some experiments cells were treated with the nucleoside reverse transcriptase inhibitor azidothymidine (0.1 μM). In experiments designed to detect transfer of HIV-1, blood or thymic DC were pulsed with virus for 2 h as described above and following 24 hours of culture, unstimulated mock infected PBMC or CD3^hi^/CD3^lo ^thymocytes were added to blood or thymic DC respectively. Cells were harvested 5 days post infection and the number of productively infected (EGFP^+^) cells detected using flow cytometry. To confirm transfer, in some experiments the pDC-CD3^hi ^thymocyte co-cultures were additionally immunostained with anti-CD3-PE at day 5 post infection.

### Immunohistochemistry

Sections (5 μm) of cryopreserved OCT-embedded thymus fragments were analysed by immunohistochemistry. All incubations were performed at room temperature in a humidified chamber. The sections were exposed to 0.3% hydrogen peroxide solution to neutralize endogenous peroxidases and then incubated with blocking buffer (10% normal goats' or fetal bovine serum) for 15 min followed with IgG1 or the mAbs; CD83, CD40 (diluted 1:200; AbD Serotec, Raleigh, NC), CD123 (diluted 1:30; BD Biosciences), HLA-DR (diluted 1:160), p24 (diluted 1:200) and CD68 (diluted 1:400; Dako, Glostrup, Denmark) for 1 h. After rinsing in PBS, the sections were exposed to biotinylated-mAb (Vectastain, Vector Laboratories Inc., Burlingame, CA) for 30 min, rinsed with PBS and then incubated with Steptavidin-HRP (Dako) for 30 min. Immunostaining was revealed using 3,3'-Diaminobenzidine substrate solution according to the manufacturer's guidelines (Dako). Sections were counterstained with haematoxylin and blued with Scott's tap water to enhance nuclear definition. Finally, sections were dehydrated through 4 changes of alcohol (70%, 95% and 2 × 100%), cleared in 3 changes of xylene and mounted with DePeX (Merck, Darmstadt, Germany).

### Flow cytometry

Flow cytometry was performed using a FACSCalibur (Becton Dickinson) and results were analysed with Weasel software (Walter and Elisa Hall Institute, Melbourne, Australia).

### Statistical analysis

Statistical analyses were performed with the Wilcoxon paired sign rank sum test or Mann Whitney test using GraphPad Prism (GraphPad software, La Jolla, CA). A p value of less than 0.05 was considered significant.

## Competing interests

The authors declare that they have no competing interests.

## Authors' contributions

VAE participated in the design of the study, performed most experiments, did the statistical analysis, and drafted the manuscript. LL performed the immunohistochemistry studies. RA provided the HIV-1 infected thymus blocks from SCID-hu-thy-liv mice infected in his laboratory. AS prepared viral stocks. EW provided antibodies for the immunohistochemistry and participated in the design of the study. SRL participated in the design and coordination of the study and revised the manuscript. PUC participated in the design and coordination of the study and revised the manuscript. All authors read and approved the final manuscript.
